# A Chinese premature infant with cleidocranial dysplasia characterized by heterozygous RUNX2 mutation and cerebral infarction: a case report

**DOI:** 10.3389/fped.2025.1643266

**Published:** 2025-09-15

**Authors:** Wuyun Zhao, Yanbin An, Xiaoyan Zhang, Na Zhuo, Cheng Cai, Gaowa Arigong

**Affiliations:** ^1^ Department of Neonatology, Inner Mongolia Maternity and Child Health Care Hospital, Hohhot, Inner Mongolia Autonomous Region, China; ^2^ Department of Neonatology, Shanghai Children’s Hospital, School of Medicine, Shanghai Jiao Tong University, Shanghai, China

**Keywords:** case report, premature infant, cleidocranial dysplasia (CCD), RUNX2, genetic

## Abstract

**Introduction:**

Cleidocranial dysplasia (CCD) is a rare genetic skeletal disorder with a low incidence rate. This study reports the first documented case in Inner Mongolia, involving a premature male newborn with a history of birth asphyxia and cerebral infarction. The patient exhibited characteristic clinical features, including abnormal cranial suture palpation, hypertelorism, depressed nasal bridge, and shortened limbs, leading to genetic testing that identified a heterozygous mutation in the RUNX2 gene—the known cause of CCD.

**Methods:**

Clinical examination included detailed physical assessment, cranial MRI, CT scan with 3D reconstruction, and chest X-ray. Genetic analysis was performed to detect mutations in the RUNX2 gene. Imaging results revealed significantly widened cranial sutures, open fontanelles, partial skull defects, right clavicular hypoplasia, and reduced bone density.

**Conclusion:**

Genetic testing confirmed a heterozygous pathogenic variant in the RUNX2 gene, consistent with a diagnosis of cleidocranial dysplasia. This case represents the first reported instance of CCD in Inner Mongolia.

**Discussion:**

This case underscores the importance of recognizing the clinical and genetic features of RUNX2-related CCD to facilitate early and accurate diagnosis. The findings aim to enhance clinical awareness of this rare condition, particularly in regions where it has not been previously reported, and to promote timely intervention and genetic counseling.

## 1 Introduction

Runt-related transcription factor 2 (RUNX2) is a critical regulator of osteoblast differentiation, it exhibits weak expression in undifferentiated mesenchymal cells, undergoes upregulation during preosteoblastic commitment, and reaches peak expression levels in immature osteoblasts, Located on chromosome 6p21 (1, 2), RUNX2 mutations are associated with cleidocranial dysplasia (CCD) (3, 4), a rare autosomal dominant skeletal disorder with an estimated prevalence of 1 in 1,000,000 (5). CCD is characterized by defective intramembranous ossification, leading to hypoplastic or absent clavicles, delayed closure of cranial sutures, wide fontanelles, dental anomalies, short stature, and skeletal abnormalities such as pubic symphysis dysplasia and scoliosis (6, 7). Additional systemic manifestations include hearing loss and sinus hypoplasia. Diagnosis relies on clinical features, imaging, and genetic testing, with management focusing on symptomatic care.

## 2 Case presentation

**Figure 1:**
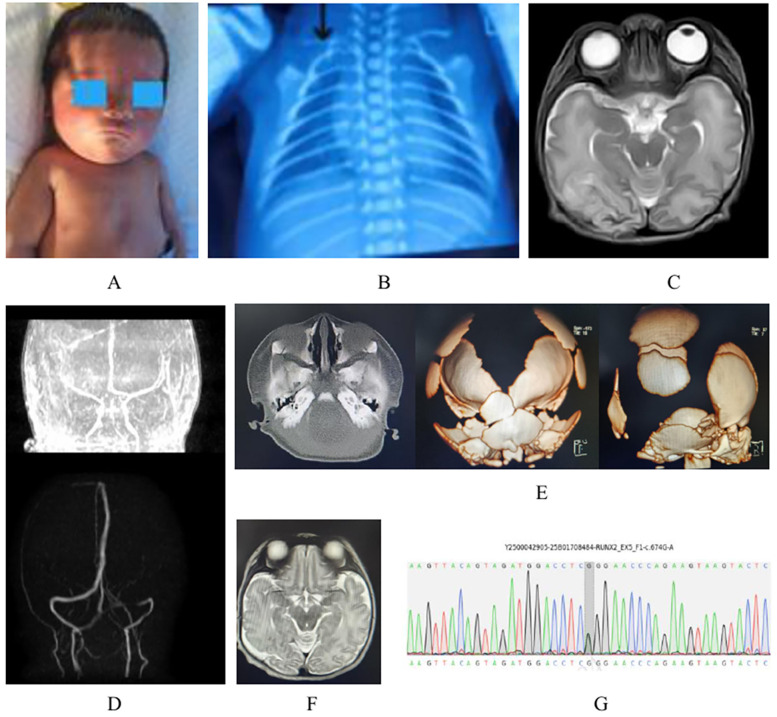
**(A)** The patient at his age 7 days. **(B)** Initial chest x-ray obtained on admission. **(C)** Brain MRI findings at 1 week of life: Cerebral infarction and hemorrhagic **(D)** Cerebral arterial (MRA) and venous (MRV) imaging at 1 week of life. **(E)** Nasal bone CT and cranial CT reconstruction. **(F)** Follow-up brain MRI at 42 days of life. **(G)** Genetic testing results of the proband (RUNX2 variant).

A male infant was admitted to the neonatal intensive care unit (NICU) at 33^+6^ weeks’ gestation with a history of resuscitation following asphyxia 15 min after birth. The infant, classified as first pregnancy, first delivery, was delivered via cesarean section at the Inner Mongolia Maternal and Child Health Care Hospital due to transverse lie. The amniotic fluid was clear, and no abnormalities were noted in the placenta, umbilical cord, or membranes. Apgar scores were 7 at 1 min (deductions for respiration, skin color, and muscle tone) and 9 at 5 min (deduction for skin color). Post-resuscitation, the infant was transferred to the NICU with diagnoses of mild asphyxia, prematurity, and neonatal grunting. Admission Physical Examination, Vital signs: Temperature (T): 36.1°C, heart rate (P): 150 bpm, respiratory rate (R): 61 breaths/min, blood pressure (BP): 60/33 mmHg, peripheral oxygen saturation (SpO₂): 80%. Anthropometrics: Length: 48 cm, head circumference: 32 cm. General appearance: Premature facies, cyanosis, hypertelorism, flattened nasal bridge, normally positioned ears, and diffuse scalp edema. Cranial examination: Poorly palpable cranial bones, widely separated sutures, and an open anterior fontanelle extending to the mid-forehead. No tenderness was elicited. Other findings: Pink skin, normal cardiac and abdominal examinations, generalized hypotonia, mild limb shortening, and webbed penis (Figure 1A). Family History: The parents of the infant were non-consanguineous, and there was no significant medical history within three generations of the family. Maternal Pregnancy History: Hypothyroidism during pregnancy: Managed with oral levothyroxine until delivery. Gestational diabetes mellitus (GDM): Diagnosed at 26 weeks of gestation, with suboptimal dietary control.

Prenatal imaging: Level III ultrasound at 26 weeks revealed poor visualization of the right fetal nasal bone, suggestive of unilateral nasal bone hypoplasia. Non-invasive prenatal testing (NIPT) was negative for common chromosomal abnormalities. Ultrasound at 29 weeks identified renal pelvic dilatation.

### 2.1 Ancillary tests

Laboratory Findings: Umbilical cord blood gas analysis: Normal. Arterial blood gas (ABG) on admission: pH 7.28, pCO₂ 52 mmHg, pO₂40 mmHg, HCO₃⁻ 24.4 mmol/L, (BE)—2.3 mmol/L, K⁺: 4.3 mmol/L, Na⁺: 132 mmol/L, Ca²⁺: 1.49 mmol/L. Glucose: 0.9 mmol/L (capillary blood glucose: 2.5 mmol/L), Lac: 2.2 mmol/L. Ionized calcium: 1.58–1.73 mmol/L, phosphate: 2.4 mmol/L. Alkaline phosphatase (ALP): 105.00 U/L. Parathyroid hormone (PTH): 4.47 pmol/L.

25-hydroxyvitamin D: 23.57 nmol/L. Coagulation profile: Mild abnormalities.

Imaging Findings: Chest x-ray on admission: Slightly reduced translucency in both lung fields. Scattered patchy and ground-glass opacities with blurred margins. Visualization of the right horizontal fissure. Right clavicle: Thinner and shorter compared to the left, with reduced bone density (Figure 1B). Brain MRI: Abnormal signal in the right parieto-occipital lobe, suggestive of cerebral infarction with hemorrhagic transformation (Figure 1C). Bilateral globus pallidus abnormalities.Mild widening of the bilateral frontotemporal extracerebral spaces. Cavum septum pellucidum. Cranial MRV: Persistent occipital sinus. Cranial MRA: Hypoplasia of the right anterior cerebral artery, suggestive of congenital variation (Figure 1D). Nasal bone CT: Thickened mucosal lining of the bilateral maxillary, ethmoid, and sphenoid sinuses. Non-fusion of bilateral nasal bones. Skull CT: Reduced white matter density. Developmental skull anomalies (Figure 1E).

Ultrasonography and Electroencephalography (EEG) Findings: Cardiac ultrasound: Patent foramen ovale (PFO). Patent ductus arteriosus (PDA). Renal ultrasound: Bilateral renal pelvic dilatation. Cranial ultrasound: Possible small subependymal hemorrhages bilaterally. Thyroid ultrasound: Normal findings.

Electroencephalography (EEG): First EEG: Abnormal waves and sporadic spikes in the bilateral temporal and central regions. Second EEG: Low-voltage activity, reduced background rhythms, scattered slow waves in bilateral temporal regions, and sporadic sharp waves predominantly in the left occipitotemporal region. Fundus screening: Retinopathy of prematurity (ROP) classified as zone 3, stage I (progressing to stage II on follow-up). Auditory evaluations: Auditory brainstem response (ABR): Bilateral failure. Transient evoked otoacoustic emissions (TEOAE): Failure in the left ear.

Whole-Exome Sequencing Methodology: Peripheral blood samples were collected for genomic DNA extraction. The DNA was fragmented, and libraries were prepared using the Roche KAPA HyperExome capture kit to enrich exonic regions and adjacent splice sites. Sequencing was performed on the MGISEQ-2000 platform.

Quality Control Metrics: Average sequencing depth in target regions: ≥200×.

Proportion of target regions with coverage >20×: >98.5%.Data Analysis: Sequencing reads were aligned to the hg19 human reference genome using Burrows-Wheeler Aligner (BWA) (8), followed by duplicate removal. GATK was utilized for base quality score recalibration, single nucleotide variant (SNV) and insertion/deletion (INDEL) calling, and genotyping. ExomeDepth (9) was employed for exon-level copy number variation (CNV) detection. Variant pathogenicity was classified according to guidelines from the American College of Medical Genetics and Genomics (ACMG) and the Association for Molecular Pathology (AMP), with refinements from the ClinGen Sequence Variant Interpretation Working Group and the Association for Clinical Genomic Science (ACGS). Results: A heterozygous pathogenic variant, c.674G>A (p.Arg225Gln), was identified in the RUNX2 gene. This variant was classified as pathogenic under ACMG criteria (Figure 1G) and partially correlated with the patient's phenotype.

Clinical Course and Management: During hospitalization, the infant received non-invasive ventilatory support, intravenous nutrition, and subcutaneous low molecular weight heparin sodium. Enteral feeding was gradually increased until full enteral feeding was achieved. By the 18th day of life, the infant's condition stabilized, and he was discharged in stable condition.

Follow-Up at 42 Days Post-Discharge: Physical examination revealed persistent cranial underdevelopment with no significant change in anterior fontanelle size compared to birth. Tandem mass spectrometry and newborn screening returned negative results. Repeat Brain MRI: Right temporo-occipital junction: Linear abnormal signal suggestive of possible schizencephaly. Previous findings: Hemorrhage in this region had resolved. Bilateral globus pallidus abnormalities were no longer evident.

Additional observations: Mildly widened bilateral temporal extracerebral spaces (Figure 1F).

## 3 Discussion

The infant's clinical features included delayed closure of the frontal and cranial sutures, widened fontanelles, hypertelorism, flattened nasal bridge, and clavicular hypoplasia. Cranial CT demonstrated abnormal skull development, while genetic sequencing identified a heterozygous G-to-A transition at nucleotide position 674 in the RUNX2 exon (c.674G>A). This variant results in the substitution of arginine by glutamine at amino acid position 225 (p.Arg225Gln) within the C-terminal runt homology domain. The loss of the positively charged arginine residue destabilizes the protein structure and disrupts its interaction with other transcription factors (10), confirming the diagnosis of cleidocranial dysplasia (CCD) secondary to a heterozygous RUNX2 mutation.

Clinical Management and Multidisciplinary Consultations: The infant, a critically ill premature neonate with complex conditions, underwent teleconsultation and multidisciplinary evaluations involving experts from the National Center for Children's Health (Beijing Children's Hospital). Recommendations included: Neonatology: Complete TORCH screening to exclude intrauterine infections contributing to cerebral infarction. Differential diagnoses considered CHARGE syndrome, Smith-Lemli-Opitz syndrome (SLOS), and chromosomal microdeletions. Neuroimaging consultation: Right occipital cytotoxic edema with irregular linear/swirl-pattern SWI hypointensities in the involved parenchyma and subcortical regions. Ventricular microcysts, suggesting possible hypoglycemic encephalopathy with hemorrhage or coexisting vascular malformations. Cranial CT: No acute hemorrhage or calcifications; incomplete membranous skull ossification. Subsequent Workup: TORCH panel: Negative. Genetic testing: Confirmed cleidocranial dysplasia (CCD). Multidisciplinary Recommendations: Metabolic Genetics (Shanghai Children's Hospital, Shanghai Jiao Tong University, School of Medicine): The identified RUNX2 variant is pathogenic. Further phenotypic evaluation for clavicular aplasia/hypoplasia, brachydactyly, and other skeletal anomalies. Pediatric Neurosurgery: Significant cranial defects warrant regular monitoring of skull development; no surgical intervention currently indicated. Pediatric Orthopedics: Surveillance for hip dysplasia and scoliosis; clavicular anomalies typically require no intervention. Neonatology: Nutritional optimization: Breast milk supplemented with human milk fortifier or preterm formula. Supplementation: Vitamin AD, vitamin D, and calcium gluconate. Follow-up: Schedule regular assessments and pursue familial verification and high-resolution chromosomal analysis.

This study has several limitations. Although the diagnosis of cleidocranial dysplasia (CCD) was confirmed in this infant, who also presented with limb shortening, the following evaluations are warranted to strengthen clinical evidence and guide management: Familial verification and high-resolution chromosomal analysis. Dental Implications: CCD frequently involves dental anomalies, such as impacted teeth. Literature suggests that surgical exposure with orthodontic traction is effective for managing impacted teeth in CCD, though outcomes depend on patient age (11). Given the genetic phenotype of this case, midline pseudocleft palate (as reported in similar cases (12) should also be considered.

The RUNX family comprises three members (RUNX1-3): RUNX1: Primarily regulates hematopoiesis (1). RUNX3: Associated with neurological and immune systems. RUNX2: A key transcriptional regulator of osteoblast differentiation, governing both intramembranous and endochondral ossification. Systemic defects in these processes underlie skeletal abnormalities in CCD (13). Mechanism of CCD: CCD arises from dysregulated RUNX2 activity during chondrocyte maturation, characterized by altered chondrocyte hypertrophy and downregulation of critical RUNX2 transcriptional targets. This disrupts endochondral ossification, leading to CCD phenotypes (14). Genotype-Phenotype Correlations: Homozygous mutations: Typically lethal in murine models due to osteogenic failure and postnatal respiratory insufficiency. Heterozygous mutations: Exhibit variable clinical manifestations, ranging from mild to severe CCD (15). RUNX2 variants: Over 200 reported, with those in the runt homology domain (RHD) linked to classic CCD features (e.g., clavicular hypoplasia, delayed fontanelle closure, dental anomalies). Variants outside the RHD often correlate with milder phenotypes (16). Expanding Phenotypic Spectrum: A study matching this patient's genotype reported extraskeletal manifestations, including limb-girdle myopathy—a rare CCD subtype (17). This highlights the need for individualized, multidisciplinary care and long-term follow-up. Patients with significant cranial defects require head protection to mitigate trauma risks (18).

## 4 Conclusion

We report a preterm infant with cleidocranial dysplasia (CCD), in whom the identified RUNX2 genetic variant aligns with previously reported pathogenic loci (19). For such cases, comprehensive genetic testing and familial verification are critical to provide actionable insights for prenatal counseling and informed reproductive planning, ultimately advancing efforts in prevention and management of genetic disorders.

## Data Availability

The datasets presented in this study can be found in online repositories. The names of the repository/repositories and accession number(s) can be found below: https://www.ncbi.nlm.nih.gov/.
